# Management of severe ARDS due to SARS-CoV-2 pneumonia using low-flow extracorporeal CO_2_ extraction

**DOI:** 10.1186/s13054-021-03777-1

**Published:** 2021-10-18

**Authors:** Rosario Molina Lobo, Beatriz Lobo-Valbuena, Federico Gordo

**Affiliations:** 1 Intensive Care Unit. Hospital Universitario del Henares, Coslada-Madrid, Spain; 2 Grupo de Investigación en Patología Crítica, Facultad de Ciencias de La Salud, Universidad Francisco de Vitoria, Pozuelo de Alarcón, Madrid, Spain; 3 Intensive Care Unit Head of Department, Hospital Universitario del Henares, Coslada, Madrid, Spain

**Keywords:** ARDS, Coronavirus infection, Respiratory failure, Extracorporeal circulation, Patient outcome assessment

Extracorporeal CO_2_ extraction (ECCO_2_R) is a technique that uses an extracorporeal device to extract excess CO_2_ from the blood by passing it through an air exchange membrane using low blood flows and requiring systemic anticoagulation for its use. The main technical difference with extracorporeal membrane oxygenation system (ECMO) is the reduced blood flow (between 300 and 500 ml/min), achieving the elimination of most of the CO_2_ produced by metabolism, thanks to the greater solubility of this gas in plasma.

This technique emerged from the need to decouple the oxygenation support from the ventilation support provided by ECMO with the main objective of optimising lung protection during mechanical ventilation (MV) [1]. There is currently no strong evidence regarding the benefit provided by the use of ECCO_2_R in this clinical context [2].

We present a series of 8 cases of patients with severe global respiratory failure due to COVID-19, admitted in ICU between December 2020 and May 2021. Regarding patients’ characteristics, we highlight mean values of ICU stay of 45.8 days (± standard deviation 15.8), PaFi of 75.1 (± 10.4) and pH 7.25 (± 0.03), despite endotracheal intubation (ETI) and protective MV with Pplat 29.1 (± 1.61). Prone position was necessary in all cases, both before and after ECCO_2_R placement. The mean number of days in prone position prior to ECCO_2_R was 3.75 days (± 1.08). Mean number of days from the start of ETI to the start of ECCO_2_R was 14.5 days, PaO2/FiO2 of 75.1 and SOFA score 6.6. Transfer to ECMO was not possible in any of the 8 cases, following to the current recommendations of the receiving centres [3].

All cases had positive SARS-CoV-2 polymerase-chain-reaction detection and severe respiratory failure without severe dysfunction of other organs or documented infections.

During ICU admission, selected patients presented persistent hypoxaemia and hypercapnia refractory to lung protective measures (TV 6–8 ml/kg, Pplat < 30) and proning. ECCO_2_R support was considered then, preserving the level of lung protection and correcting the expected deterioration of ventilation with worsening respiratory acidosis obtained after achieving an increase in PEEP. Our objectives were also to decrease Pplat in a range of less than 28, to decrease respiratory rate under 25 bpm and to decrease TV (under 7 ml/kg).

Table 1 shows the respiratory status prior to initiation of therapy, and Table 2 shows the results obtained 12–24 h after initiation of the ECCO_2_R.

**Table 1:** Characteristics

N	Sex (F/M)	Age (yr)	BMI (kg/m^2^)	Days Symp/ECCO_2_R	Days EIT/ECCO_2_R	Compliance (ml/cmH_2_O)	PO_2_/FiO_2_	Days on ECCO_2_R	ICU LOS after ECCO_2_R	Out
1	F	53	33	26	15	17	64	5	47	a
2	F	55	37	25	11	13	70	7	33	a
3	M	74	28	53	13	34	96	1	24	d
4	M	67	33	29	19	8	71	6	23	d
5	F	61	32	16	21	28	61	4	12	a
6	M	69	36	20	9	42	78	3	20	d
7	M	69	36	23	14	35	81	5	8	a
8	M	64	25	23	14	14	80	8	1	d

N = Number of cases, F = female, M = male, Yr = years, BMI = body mass index, Days Symp/ECCO_2_R = number of days from symptom onset to ECCO_2_R placement, Days EIT/ECCO_2_R = number of days from intubation to ECCO_2_R placement, LOS = length of stay after ECCO_2_R, Out. = outcome, a = alive, d = death

**Table 2:** Data before and after 24-h initiating ECCO_2_R

	Before ECCO_2_R						After ECCO_2_R and PEEP trial					
N	TV (ml/kg)	Pplat (cmH_2_O)	DP (cmH_2_O)	PEEP (cmH_2_O)	pCO_2_	FiO_2_	TV (ml/kg)	Pplat (cmH_2_O)	DP (cmH_2_O)	PEEP (cmH_2_O)	pCO_2_	FiO_2_
1	6	32	24	8	75	0.8	4.5	30	16	14	63	0.6
2	6.4	27	16	6	71	1	4.5	23	10	10	59	0.75
3	7.2	28	20	8	81	1	5	28	18	10	67	0.85
4	7.8	31	18	12	65	1	4.6	28	13	14	57	0.9
5	5.6	28	16	12	84	1	5	28	10	18	51	0.7
6	6.7	28	11	14	66	0.9	6.5	25	9	16	51	0.7
7	5.1	29	17	12	87	1	4	28	12	16	64	0.65
8	6	30	18	8-	77	80	4.2	26	14	12	62	0.75

N = Number of cases, TV = tidal volume, DP = driving pressure

The therapeutic objectives of ProLUNG® (blood flow 400 ml/min and air flow 15 L/min), with a rise in pH (7.32–7.35), were achieved in the first 12 h of treatment. Results are shown in Table 2. Regarding technical complications, thrombocytopenia not induced by heparin was observed in one of the cases, requiring withdrawal of treatment after 5 days.

Regarding the outcome data on the ECCO_2_R device, the mean duration of therapy was 5.25 days, with a mean blood flow of 400 ml/min and air flow of 12 lpm. Fifty percentage of the patients survived, with an ICU discharge of 25 days (average value) after disconnection from the ECCO_2_R. Case number 8 died within a day after removal of the CO_2_ extractor.

These cases, as in previous studies, show us the possible usefulness of applying ECCO_2_R based on individual clinical and physiological criteria [4], without development of serious complications, although other known published series report relevant complications [5].

ECCO_2_R system could be applied as a tool to optimise the degree of lung protection, essential in patients with severe ARDS. In our experience, ECCO_2_R is a simple and feasible therapeutic option for patients with severe respiratory diseases in whom conventional treatment has been maximised. Further studies are needed to strengthen the scientific evidence in this context [6].

## Data Availability

All data generated or analysed during this study are included in this published article.
